# A phosphodiesterase-4 inhibitor reduces lung inflammation and fibrosis in a hamster model of SARS-CoV-2 infection

**DOI:** 10.3389/fimmu.2023.1270414

**Published:** 2023-10-02

**Authors:** Afsal Kolloli, Santhamani Ramasamy, Ranjeet Kumar, Annuurun Nisa, Gilla Kaplan, Selvakumar Subbian

**Affiliations:** ^1^ Public Health Research Institute (PHRI) at New Jersey Medical School, Rutgers, The State University of New Jersey, Newark, NJ, United States; ^2^ University of Cape Town, Cape Town, South Africa

**Keywords:** COVID-19, inflammation, cytokine storm, host-directed therapy, lung function, fibrosis, phosphodiesterase-4 inhibitor, hamster

## Abstract

**Introduction:**

The Severe Acute Respiratory Syndrome-Coronavirus-2 (SARS-CoV-2) infection involves pulmonary inflammation that can progress to acute respiratory distress syndrome, a primary cause of lung damage/fibrosis in patients with Coronavirus Disease-2019 (COVID-19). Currently, there is no efficacious therapy available to alleviate lung fibrosis in COVID-19 cases. In this proof-of-concept study, we evaluated the effect of CC-11050, a small molecule phosphodiesterase-4 inhibitor, in dampening lung inflammation and fibrosis in a hamster model of SARS-CoV-2 infection.

**Methods:**

Following intranasal inoculation with SARS-CoV-2/WA- 1/2000 strain, hamsters were treated with CC-11050 or placebo by gavage from day-1 until day-16 post-infection (dpi). Animals were monitored for body weight changes, virus titers, histopathology, fibrotic remodeling, cellular composition in the lungs between 2 and 16 dpi.

**Results:**

We observed significant reduction in lung viral titer with concomitant reduction in inflammation and fibrotic remodeling in CC-11050 treated hamsters compared to untreated animals. The reductions in immunopathologic manifestations were associated with significant downregulation of inflammatory and fibrotic remodeling gene expression, reduced infiltration of activated monocytes, granulocytes, and reticular fibroblasts in CC-11050 treated animals. Cellular studies indicate a link between TNF-α and fibrotic remodeling during CC-11050 therapy.

**Discussion:**

These findings suggest that CC-11050 may be a potential host-directed therapy to dampen inflammation and fibrosis in COVID-19 cases.

## Introduction

Coronavirus disease-2019 (COVID-19), caused by the severe acute respiratory syndrome coronavirus-2 (SARS-CoV-2), is a major global health concern that affected about 770.5 million people and caused 6.9 million deaths worldwide by September 11, 2023 (1). In humans, symptomatic SARS-CoV-2 infection is often associated with atypical pneumonia and hyper-inflammation that can progress to acute respiratory distress syndrome (ARDS), a life-threatening complication in COVID-19 patients (2, 3). With no efficacious treatment available to manage ARDS, a majority of severe COVID-19 cases are treated with different types of antiviral drugs including Remdesivir or Paxlovid together with corticosteroids (4).

Severe pulmonary disease symptoms of COVID-19, which are a consequence of tissue damage due to an exacerbated inflammatory response, are marked by elevated levels of TNF-α, IL-1β, and other inflammatory mediators (3, 5). In addition, acute SARS-CoV-2 infections induce the production of IL-6 and TGF-β, which are profibrotic agents that augment fibroblast differentiation into myofibroblasts, and stimulate matrix metalloprotease (MMP)-mediated collagen production/deposition that contributes to lung fibrosis (3, 6). Indeed, clinical studies have reported that about a third of COVID-19 cases developed fibrotic abnormalities that significantly impacted the quality of life in these patients (7). Since an exacerbated inflammatory response is a major cause of disease pathology and fibrosis among COVID-19 cases, the inclusion of anti-inflammatory drugs, such as corticosteroids may be beneficial for the better management of the disease. However, treatment with corticosteroids poses a high risk of immunosuppression, which would increase the patient’s vulnerability to acquiring other infectious diseases, including tuberculosis (TB) (8). Therefore, the development and evaluation of more efficacious anti-inflammatory/immunomodulatory drugs that do not cause immunosuppression are urgently needed to dampen the inflammation and lung fibrosis associated with COVID-19.

Phosphodiesterase-4 inhibitors (PDE4i) are a class of anti-inflammatory immunomodulators that show beneficial effects as adjunct therapeutic drugs, to control inflammation (9–12). PDE4i treatment was shown to significantly dampen the production of pro-inflammatory cytokines, chemokines, and matrix metalloproteinases, and reduce lung fibrosis in mouse and rabbit models of TB (10, 13–17). In a clinical trial, adjunctive therapy with the PDE4i CC-11050 resulted in significant improvement in the lung function of patients treated with anti-TB drugs (9). These findings strongly suggest that the PDE4i may be a promising drug to control immunopathology and prevent/reduce fibrosis during SARS-CoV-2 infection and for better management of post-COVID-19 complications (7). In the present study, we investigated the effect of treatment with CC-11050 on lung inflammation and fibrotic remodeling during pulmonary SARS-CoV-2 infection using a hamster model.

## Methods

### Ethical statement

All the experiments involving infectious SARS-CoV-2 were conducted in Biosafety level 3 facilities at the Public Health Research Institute of Rutgers University as per approved standard operating procedures. All animal procedures were performed in bio-safety level 3 facilities following procedures approved by the Rutgers University Institutional Animal Care and Use Committee (#PROTO202000103), which is consistent with the policies of the American Veterinary Medical Association (AVMA), the Center for Disease Control (CDC), and the United States Department of Agriculture (USDA).

### Virus propagation and infectivity titration

SARS-CoV-2 (strain USA-WA1/2020) was obtained from BEI Resources (ATCC, Manassas, VA, USA). A viral plaque-forming unit (PFU) assay was performed to quantitate live viruses in the inoculum and infected tissue homogenates. Virus propagation, titration, infectivity assays, and antibody titration were performed using Vero E6 cells (ATCC, Manassas, MA, USA) as described previously (18). Briefly, for virus propagation, Vero E6 cell monolayer was infected with SARS-CoV-2 at a multiplicity of infection (MOI) of 0.2 in DMEM media supplemented with 2% FBS (ThermoFisher Scientific, Waltham, MA, USA). After 48 hours of infection, the supernatant from infected cells was collected. For the PFU assay, Vero E6 cells were seeded at 5x10^5^ cells per well onto 6-well plates and incubated for 18-24 hours. Virus-containing supernatant was serially diluted 10-fold from 10^-2^ to 10^-6^. The spent media from Vero E6 cells (in 6-well plates) was replaced with diluted virus media at 400µL/well and incubated at 37˚C for 1 hour. Unattached viruses were then removed by gentle aspiration of the media, and the infected monolayers were overlaid with a mixture containing equal amounts of 2X MEM (ThermoFisher Scientific, Waltham, MA, USA) with 8% FBS and 1.6% pre-melted agarose (VWR International, Radnor, PA, USA). The plates were incubated at 37˚C for 72 hours and plaques were visualized by staining with 0.2% crystal violet (Sigma-Aldrich, St. Louis, MO, USA).

Unless specified, all chemicals and reagents were purchased from Sigma-Aldrich.

### Golden Syrian hamster infection and CC-11050 treatment

Forty (n=40) male Golden Syrian hamsters (*Mesocricetus auratus*) of 6-8 weeks were purchased from Envigo Corporation, Denver, PA, USA, and housed as two animals per cage. Male animals were relatively less aggressive, easy to handle, and could be housed together, which is critical for the BSL3 housing of these animals. Food and water were given ad libitum throughout the experiment. Animals were acclimatized for seven days in the BSL3 facilities before being allocated to experiments. Bodyweight, food and water intake were monitored daily for each animal throughout the experiment. Hamsters were randomly allocated into groups and infected with SARS-CoV-2 (10^2.5^ PFU) in 50µL of sterile 1X PBS via intranasal inoculation as described earlier (18). CC-11050 solution for treatment was prepared in water. A group of infected hamsters was treated with CC-11050 (n=20) at the dose of 25mg/kg in 2ml by oral gavage. Another group was treated with 2ml of water only (placebo; n=20); the treatment was started on the day of infection and continued until the end of the experiment (day-16 pi). Four hamsters each from the untreated and CC-11050 treated groups were euthanized at 2, 4-, 7-, 12- and 16-days post-infection (dpi). Blood was collected by cardiac puncture before necropsy when animals were under deep anesthesia. The lungs were harvested and weighed aseptically. A portion of the lung was transferred to 10% buffered formalin for histopathology analysis. For RNA isolation, a portion of the lung was stored in TRI reagent (Molecular Research Center Inc, Cincinnati, OH, USA). The animal group size was calculated based on our previous study on the hamster model with a 20% difference in mean with 80% power and an alpha level of 0.05 between the untreated and CC-11050 treated groups (18). Accordingly, n=4 per group per time point was used for all the *in vivo* infection and treatment experiments.

### Quantification of infectious viral titer in the lungs

Approximately 40% of each lung was transferred into DMEM containing penicillin-streptomycin and was homogenized in a Mini Bead Mill Homogenizer (VWR International, Radnor, PA, USA) for five cycles of 20 seconds each. The lung homogenates were centrifuged, and the supernatant was filtered through a 0.45 µ nylon filter (VWR International, Radnor, PA, USA). The filtrate was diluted in serum-free DMEM, and 400 µL was used to infect one-day-old Vero E6 cell monolayers in six-well plates at 37˚C for 1 hour as described previously (18). Unattached viruses were removed, and the infected monolayers were overlaid with a mixture containing equal amounts of 2X MEM with 8% FBS and 1.6% low-melting agarose. The plaques were visualized on the third day by staining with 0.2% crystal violet.

### Histopathology analysis

A portion of each lung was fixed in 10% buffered formalin, embedded in paraffin, cut into 5 microns sections and placed on positively charged glass slides. The sections were stained with hematoxylin and eosin (general histology) or Masson’s trichrome (collagen deposition/fibrosis). The histopathological examination was performed by a veterinary virologist (S.R.) using the EVOS FL Cell imaging system (ThermoFisher Scientific, Waltham, MA, USA). The pulmonary disease pathology was assessed based on the histopathology scoring (0–5), which involves the degree of edema, alveolar/bronchiolar hyperplasia, mononuclear infiltration, emphysema, bronchiolar/arteriolar smooth muscle thickening, and vascular lesions as reported previously (18). To study lung fibrosis, Masson’s trichrome stained lung sections were analyzed at 40x magnification, and various forms of fibrotic remodeling, such as thickening of the alveolar basement membrane, interstitial deposition of collagen fibers, perivascular adventitial layer proliferation, peribronchiolar fibroelastic layer proliferation, and pleural membrane thickening were scored from 0 to 5 according to the degree of collagen fiber deposition.

### Immunohistochemistry

Paraffin-embedded tissue sections of 5-micron thickness were treated with xylene and descending grades of ethanol, and antigen retrieval was performed using 10mM citrate buffer at 90˚C for 40 minutes. For hybridization with single molecule mRNA (smRNA) probes, the sections were washed with 2X SSC buffer containing 10% formamide in 2X SSC (ThermoFisher Scientific, Waltham, MA, USA). The sections were hybridized with fluorochrome-labeled smRNA probes (Biosearch Technologies, Dexter, MI, USA) against SARS-CoV-2 (TMR), MMP1 (TexasRed), TGFB1 (Cy5) and TNFA (TexasRed) in a fluorescent *in situ* hybridization (FISH) assay (sm-FISH). The sections were also stained to visualize activated macrophages (anti-IBA-1 antibody; Abcam, MA, USA), neutrophils (anti-Calprotectin antibody; Abcam, MA, USA), and fibroblasts (anti-ER-TR7 antibody; Novus Bio, CO, USA). The fibroblast was detected by staining with a secondary antibody conjugated with Alexa Fluor-647. The slides were rinsed in wash buffer, treated with TrueBlack Lipofuschin autofluorescence quencher (Biotium Inc, Fremont, CA, USA), and mounted with a coverslip. Images were captured from 6-10 fields per lung section per animal (n =3 per group per time point) using a Zeiss 200M fluorescent microscope (Carl Zeiss Microscopy LLC, White Plains, NY, USA). Each image was analyzed using ImageJ software and the number of cells positive for each marker was normalized to the total number of cells in each field containing at least 200-400 cells.

### Hydroxyproline assay

Hydroxyproline content has been used as a reliable surrogate to determine the total amount of collagen in tissues (19–21). To determine the extent of lung fibrotic remodeling, we measured the hydroxyproline content by a spectrophotometric assay using Hydroxyproline Assay Kit (BioVision, Milpitas, CA, USA). Briefly, lung tissue was homogenized in PBS (10mg/100 µl of PBS) using a bead beater. Subsequently, one volume of 12 N HCl was added to each homogenized sample in a pressure-tight, Teflon-capped vial and hydrolyzed overnight at 100°C. After hydrolysis, 10 µL of each sample was transferred to a 96-well plate and evaporated to dryness. Then, samples were oxidized with chloramine-T for 5 min at room temperature. The reaction mixture was then incubated in dimethyl amino benzaldehyde at 60°C for 90 min and cooled to room temperature. Sample absorbance was measured at 560 nm. Hydroxyproline content was estimated from the standard curve and expressed as micrograms of hydroxyproline per mg of total lung tissue.

### RNA isolation and RT PCR

Total RNA was isolated from the lungs of uninfected and SARS-CoV-2 infected hamsters with or without CC-11050 treatment using TRIreagent and purified by RNeasy mini-columns following manufacturer’s instructions (Qiagen, Valencia, CA, USA). cDNA synthesis was performed using a High-Capacity cDNA Reverse Transcription Kit with one microgram of total RNA, as per the standard protocol (ThermoFisher Scientific, Waltham, MA, USA). The expression level of CRP, TNF-α, IL-1β, IL-6, MIP-1α, IL17-RA, IP-10, CXCL12, CCR1, IL-10, IL-4, p35, MMP-2, MMP-9, MMP-13, TIMP2, TIMP3, TGF-β1, COLIA1, TAGLN, MYH11, SMAD2, GMCSF, GCSF, and FGF2 genes were measured using gene-specific primers and Power SYBR Green PCR MasterMix as per the manufacturer’s protocol (ThermoFisher Scientific, Waltham, MA, USA). The expression level of the target gene was normalized to housekeeping GAPDH level and shown as a fold change in untreated or CC-11050 treated versus uninfected hamster samples. The list of primers used in this study is provided in **Supplementary Table 1**.

### Estimation of cytokine levels in the lung homogenate

The levels of TNF-α, IL-6 and TGF-β were estimated in lung homogenates using ELISA kits as per the manufacturer’s instructions (MyBiosource, San Diego, CA, USA). Briefly, 100 mg of lung tissue was homogenized in PBS using a bead beater and then centrifuged for 5 minutes at 5000×g. Lung homogenates were diluted with sample dilution buffer at a 1:1 ratio and 100 µl of diluted samples were used to estimate the cytokine levels. Absorbance was measured at 450 nm and the cytokine level was estimated from the standard curve and the levels of TNF-α, IL-6 and TGF-β were expressed as picograms per ml of lung tissue homogenate.

### Lung transcriptome analysis

We have previously reported the RNAseq analysis of total RNA isolated from SARS-CoV-2 infected and uninfected hamster lungs (18). For the current study, we interrogated the RNAseq data (GEO accession# PRJNA846295) using Ingenuity Pathway Analysis (IPA; Qiagen, Redwood City, CA, USA) to determine the expression profile of network genes related to lung inflammation and fibrosis Partek Genomics Suite ver 6.8 (Partek Inc, Chesterfield, MO, USA) was used to construct the heat map of the network genes.

### Lung fibroblast culture and immunofluorescence staining

To evaluate the effect of CC-11050 treatment on inflammation-mediated fibroblast activation, human lung fibroblast cell line (MRC-5 cells; ATCC, Manassas, VA, USA) were co-cultured with or without supernatants from Vero E6 cells with or without SARS-CoV-2 infection and the production of alpha-smooth muscle actin (αSMA) was detected by immunofluorescence labeling.

Approximately 0.05 x 10^6^ MRC-5 cells were seeded on coverslips in 24-well plates and cultured in RPMI medium supplemented with 10% (v/v) fetal bovine serum and antibiotics mixture. Fibroblast cells were treated with TNF-α (20ng/ml, Novus Bio) or CC-11050 (50µL/ml) or both or kept untreated for 24 and 48 hr as described previously (22, 23). For immunofluorescence labeling at specific time points, cells were fixed in 4% formaldehyde solution and permeabilized in 0.1% Triton X-100 solution. Cells were blocked and labeled with primary antibody (Goat anti- αSMA antibody, Novus Biologicals, Centennial, CO, USA) and secondary antibody (anti- goat IgG conjugated Texas Red, Abcam, Boston, MA, USA). After immunofluorescence staining, coverslips were mounted on glass slides using fluoroshield mounting media with DAPI (Abcam, Boston, MA, USA). Images were captured at 20x magnification of 10 random fields per slide using a Zeiss 200M fluorescent microscope (Carl Zeiss Microscopy LLC, White Plains, NY, USA). Images were analyzed (approximately 80 to 120 cells per image x 10 different fields) using ImageJ software (NIH, Bethesda, WA, USA), and the number of αSMA+ cells was counted for all treatment conditions. The percentage of αSMA+ cells was determined by dividing the number of positive cells by the total number of cells from each field and averaging per slide.

### Statistical analysis

Statistical analysis was performed using GraphPad Prism-8 (GraphPad Software, La Jolla, CA), and values were plotted in the graphs as mean ± standard error (SE). The unpaired Student’s t-test was used to analyze the significance of data between the untreated and CC-11050 treated groups. For multiple group comparison, One-way ANOVA with Tukey’s posthoc test was used. For all the experimental comparisons between groups, a p≤ 0.05 was considered statistically significant.

### Reagent validation

The Vero E6 (RCB Cat#RCB001, RRID: CVCL0059) and the MRC-5 (ATCC Cat# CCL-171, RRID: CVCL_0440) cell lines were validated by the commercial supplier (ATCC, Manassas, VA). These cell lines were tested for mycoplasma contamination by microscopic analysis; no such contamination was observed. All the antibodies used in this study, including ELISA kits, were validated, and documented by the manufacturers of respective antibodies (IBA-1 and Calprotectin antibodies by Abcam; ER-TR-7 antibody by Novus Bio, a bio-techne brand; and ELISA antibodies by My BioSource).

## Results

### CC-11050 treatment reduced the viral titer in the lungs

**Figure 1:**
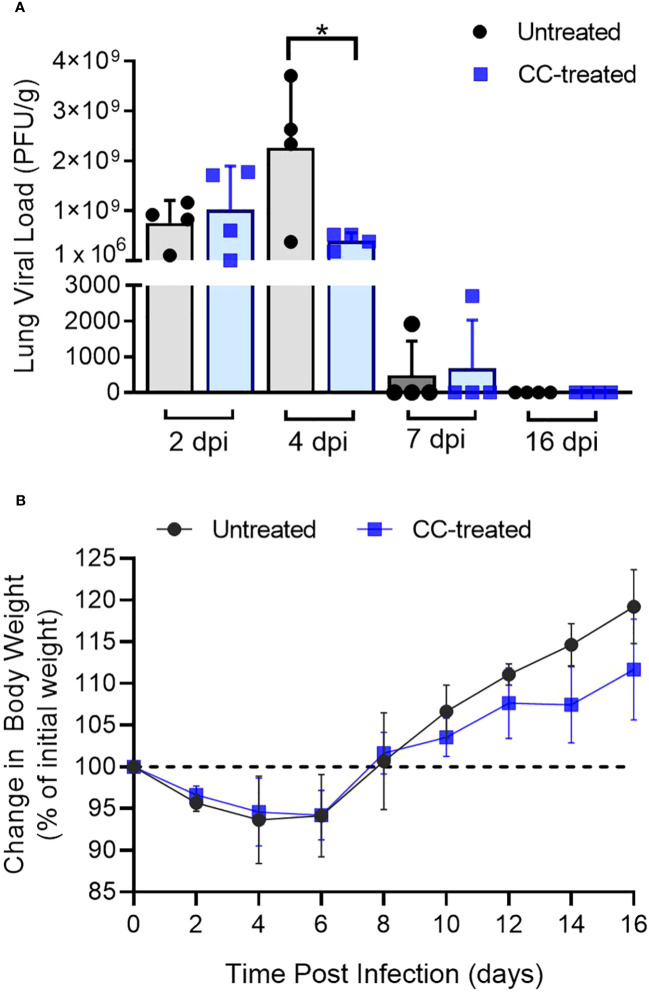
Lung viral titer and body weight change in SARS-CoV-2 infected hamsters. **(A)** Replicating viral titer in SARS-CoV-2 infected hamster lungs treated with CC-11050 (CC-treated) or no treatment (Untreated) at 2-, 4-, 7- and 16-days post-infection (dpi). The viral titer is expressed as plaque-forming units per gram (PFU/g) of lung tissues (n=4 animals per group per time point). **(B)** Percentage body weight changes compared to initial weight in Untreated and CC-treated SARS-CoV-2 infected hamsters up to 16 dpi (n=4 animals per group per time point). Values are plotted as mean ± standard error (SE). Data were analyzed by the unpaired t-test. *p<0.05.

To study the effect of CC-11050 treatment on the burden of replicative SARS-CoV-2, we performed a PFU assay on hamster lung homogenates at 2, 4, 7, 12 and 16 dpi. Infectious viruses were found in the lungs of untreated hamsters at 2, 4 and 7 dpi, with a peak viral titer at 4 dpi (**Figure 1A**). The mean infectious viral titer in the lungs at 4 dpi was significantly lower (p=0.0373) in the CC-11050 treated hamsters than the untreated controls (**Figure 1A**). At 7 dpi, low numbers of infectious SARS-CoV-2 (about 3 log_10_ PFU) were found in the lungs of one animal in both the untreated and the CC-11050 treated groups. No infectious virus was detected in the lungs of any of the infected hamsters at 12 dpi (not shown) and 16 dpi. Furthermore, we also studied the effect of CC-11050 on viral replication in the Vero E6 cell line. However, we did not find any significant difference in viral titer in CC-11050 treated Vero E6 cells as compared to untreated cells (**Supplementary Figure 1**). This result suggests that CC-11050 treatment does not have a direct antiviral effect but rather CC-11050 treatment can reduce the replicative viral titer in the lungs of SARS-CoV-2-infected hamsters during acute stages of infection (i.e. 4 dpi) through its host immune modulatory effects.

### CC-11050 treatment did not affect the body weight during infection

Change in body weight has been used as a clinical parameter of SARS-CoV-2 infection in experimental animals and humans (18, 24–28). Following pulmonary SARS-CoV-2 infection, a reduction in the body weight of untreated and CC-11050 treated hamsters was noted from 2 dpi until 6 dpi, with a peak body weight loss of about 12% noticed in both groups between 4 and 6 dpi. Starting at 8 dpi the animals gained body weight gradually until 16 dpi (**Figure 1B**). There were no significant differences in body weight change between the untreated and CC-11050 treated infected hamsters.

### CC-11050 administration ameliorated disease pathology in the lungs

**Figure 2:**
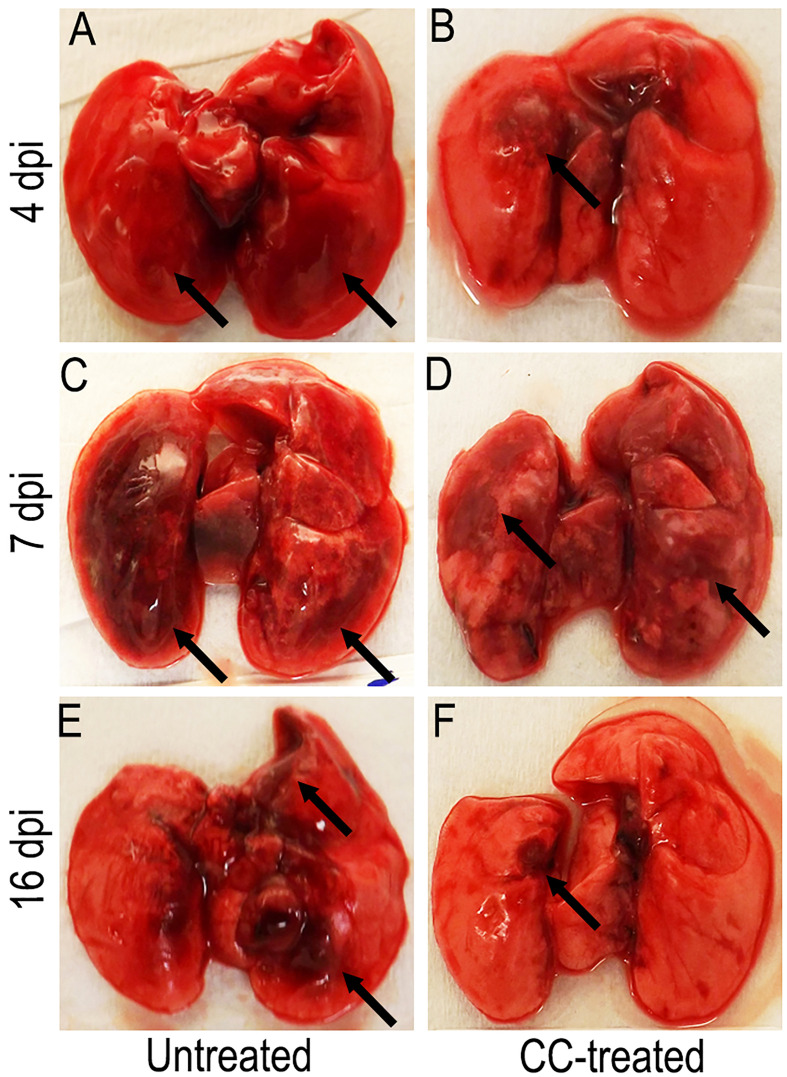
Representative gross pathology of SARS-CoV-2 infected hamster lungs with or without CC-11050 treatment. **(A)** On 4 dpi, untreated hamsters showed multifocal moderate-to- severe red hepatization (arrows) spread across ~50% of the lungs. **(B)** In CC-11050 treated group, mild-to- moderate red hepatization (arrow) was observed particularly in the right anterior lobe and left hilar region of the lungs. **(C)** On 7 dpi, severe multifocal hepatization (arrows) involving up to 80% of the lungs was noted in the untreated hamsters. **(D)** A similar hepatization pattern (arrows) was observed in CC- 11050-treated hamsters. **(E)** On 16 dpi, untreated hamster lungs had brown multifocal hepatization and emphysema (arrows). **(F)** No remarkable hepatization was noted (arrow), and the lungs appeared normal in color and consistency in CC-11050-treated hamsters. Representative images of n=4 animals per group per time point.

We next examined the gross lung pathology in SARS-CoV-2 infected hamsters with or without CC-11050 treatment. As shown in **Figure 2**, by 4 dpi the lungs of untreated hamsters had multifocal/extensive red hepatization with pleural adhesions and emphysema (**Figure 2A**). Among CC-11050 treated hamsters at 4 dpi, red hepatization was mostly observed in the right anterior lobe and the left hilar region (**Figure 2B**). In the absence of treatment, the lung pathology progressed further by 7 dpi with multifocal severe hepatization, involving extensive lung parenchyma (**Figure 2C**). A somewhat milder hepatization pattern with multifocal areas and emphysema was observed in the lungs of CC-11050 treated hamsters at 7 dpi (**Figure 2D**). At 16 dpi, moderate multifocal brown hepatization and emphysema were observed in the untreated hamster lungs (**Figure 2E**). In contrast, the lungs of CC-11050 treated hamsters appeared to have normal color and consistency with small brownish foci of resolution of pneumonia and hepatization (**Figure 2F**). Together, these observations suggest that CC-11050 treatment can accelerate the amelioration of lung pathology in SARS-CoV-2-infected hamsters.

**Figure 3:**
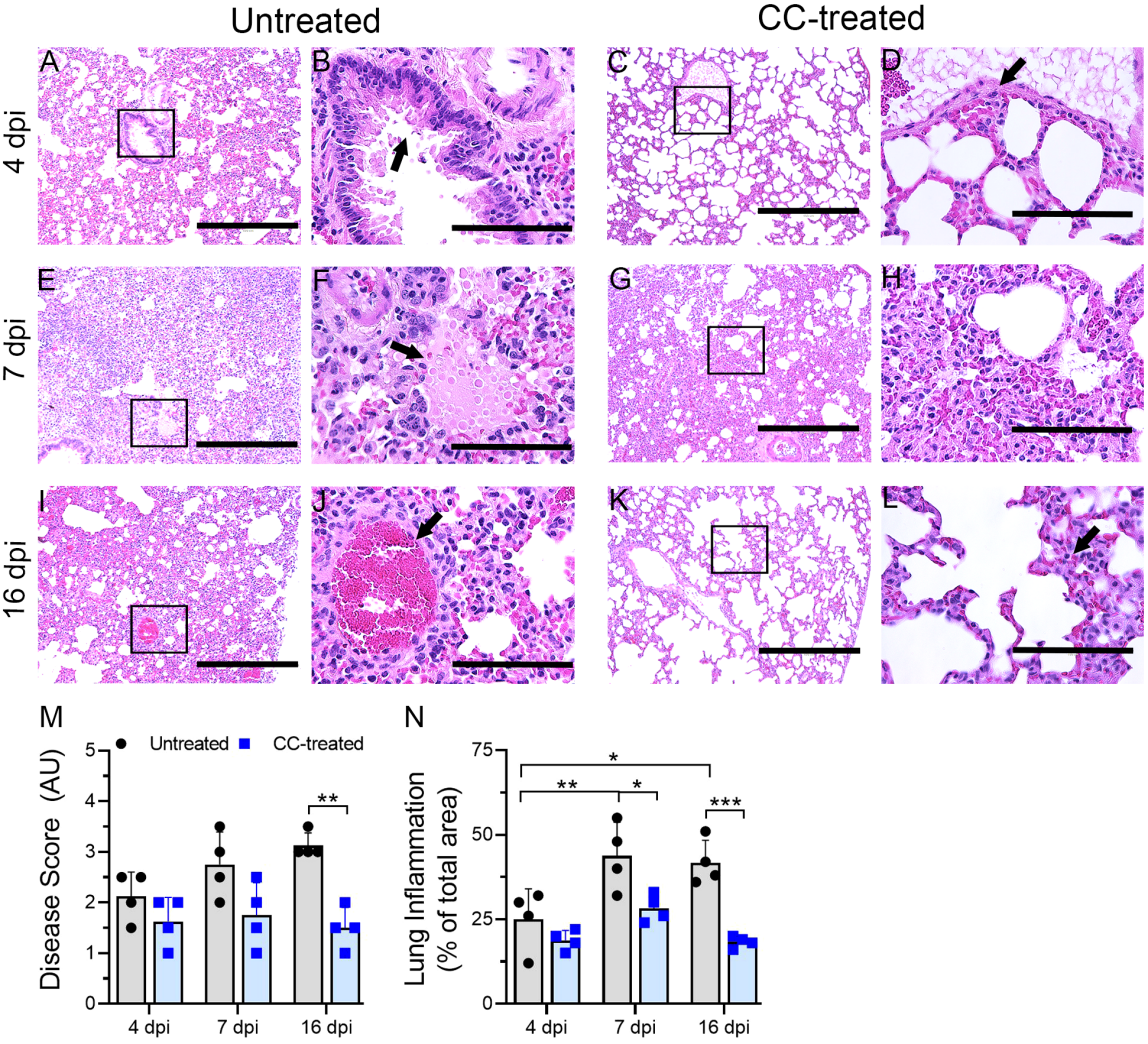
Histopathologic analysis of SARS-CoV-2 infection in untreated and CC-11050 treated hamster lungs. The untreated hamsters had moderate inflammation **(A)** and epithelial hyperplasia (arrow in **B**) on 4 dpi. In CC-11050 treated hamsters, mild infiltration of inflammatory cells **(C)** with bronchiolar epithelial hyperplasia (arrow in **D**) was noted on 4dpi. Severe infiltration of inflammatory cells **(E)** with leakage of platelets into alveoli (arrow in **F**) was noted in the untreated group on 7dpi. At this time, a moderate to severe infiltration of inflammatory cells and pulmonary edema **(G, H)** was observed in CC-11050 treated animals. At 16 dpi, a moderate inflammation involving alveolar spaces **(I)** with thrombotic occlusion (arrow in **J**) was noted in the untreated animals, while a resolution of inflammation **(K)** with obliteration of alveoli and denudation of endothelial cells in the arteriole (arrow in **L**) were noted in CC-11050 treated hamsters. Representative images **(A, C, E, G, I, K)** are 100x magnifications (scale bar 400 µm), and **(B, D, F, H, J, L)** are 400x magnifications (scale bar 100 µm) of the marked areas in **(A, C, E, G, I, K)** respectively. Lung histopathology score **(M)** was determined in SARS-CoV-2 infected hamsters with or without CC-11050 treatment at 4, 7 and 16 dpi (n=4 animals per group per time point) and presented as Disease Score (AU-arbitrary unit). Data were analyzed by One-way ANOVA (**p<0.01). Morphometry analysis **(N)** was performed to evaluate the percentage of lungs involved in inflammation relative to the total lung area at 4, 7 and 16 dpi (n=4 animals per group per time point). Data were analyzed by One-way ANOVA. * p<0.05; **p<0.01; ***p<0.005.

Lung sections from untreated and CC-11050 treated hamsters at 4, 7 and 16 dpi were used for histopathology analysis. The untreated-SARS-CoV-2 infected hamster lungs showed moderate infiltration of inflammatory cells and bronchiolitis by 4 dpi (**Figures 3A, B**). At 4 dpi the lungs of CC-11050 treated hamsters showed mild-to-moderate levels of inflammatory cell infiltration, bronchiolar epithelial hyperplasia, and vascular muscle proliferation (**Figures 3C, D**). At 7 dpi, all untreated hamster lungs showed moderate to severe infiltration of inflammatory cells, congestion of capillaries in the alveolar wall, edema, and alveolar collapse (**Figures 3E, F**). Similar disease presentation was observed in CC-11050 treated hamsters, although the extent of infiltration was mild-moderate in three hamsters and severe in only one hamster in this group (**Figures 3G, H**). By 16 dpi, the untreated hamster lungs showed a moderate cellular accumulation with less inflammation and signs of resolution of inflammation compared to 7 dpi (**Figures 3I, J**). In contrast, the inflammation was strikingly resolved in one hamster, while residual mild cellular infiltration was noted in the other three hamsters that received CC-11050 treatment (**Figures 3K, L**).

A significantly higher percentage of lung area was infiltered at 7 and 16 dpi, compared to 4 dpi in the untreated hamsters (**Figure 3N**). No significant difference in the inflammatory lung area was noted among the CC-11050 treated hamsters at 4, 7 and 16 dpi, although the percentage of the area involved was somewhat higher at 7 dpi, compared to 4 and 16 dpi (**Figure 3N**). Compared to the uninfected group, the CC-11050 treated hamster lungs had a significant reduction in the percentage of lung area involved in inflammation at 7 and 16 dpi (**Figure 3N**). A similar pattern was observed when a histopathology scoring system was used to evaluate the extent of disease in the lungs (**Figure 3M**). While the histopathology score increased over time in the untreated hamster lungs until 16 dpi, CC-11050 treatment prevented the lung disease, resulting in a significantly lower histopathology score compared to the untreated animals at 16 dpi. Taken together, the histology analysis clearly shows that CC-11050 treatment dampens lung inflammation in SARS-CoV-2 infected hamsters, particularly at later stages of infection (i.e. 7 to 16 dpi).

### CC-11050 administration decreased the interstitial collagen deposition in the lungs

**Figure 4:**
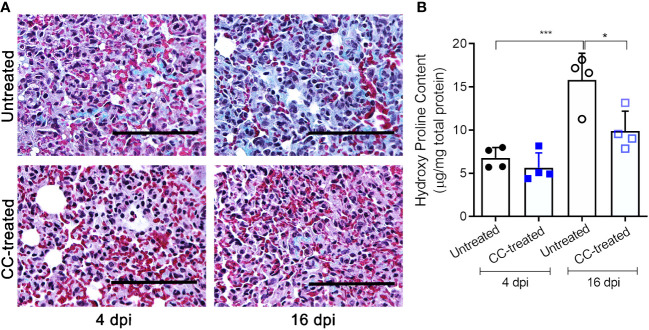
CC-11050 treatment significantly reduces collagen deposition in the SARS-CoV-2 infected hamster lungs. **(A)** Lung fibrotic remodeling (blue coloration) in SARS-CoV-2 infected hamsters with or without CC-11050 treatment was determined by Masson’s trichrome staining method followed by microscopy on 4 dpi and 16 dpi. Images were photographed at 400 × magnification (Scale bar = 100 μm). **(B)** Hydroxyproline content of the untreated and CC-11050-treated (CC-treated) hamster lungs was measured by a spectrophotometric method and represented as micrograms of hydroxyproline per mg of the lung. (n=4 animals per group per time point repeated twice). Values plotted are mean ± SE. Data were analyzed by the unpaired t-test. * p<0.05; ***p<0.005.

We next investigated the effect of CC-11050 administration on lung fibrotic remodeling in SARS-CoV-2-infected hamsters. We evaluated the fibrotic remodeling using (i) collagen staining (Masson’s trichrome staining) of lung sections, and (ii) measuring hydroxyproline content at early (4 dpi) and late (16 dpi) stages of infection. The lung sections stained for collagen were scored for different pathological forms of fibrosis such as interstitial deposition of collagen, thickening of alveolar basement membrane perivascular adventitial layer proliferation, peribronchiolar fibroelastic layer proliferation, and pleural membrane thickening (**Table 1**). Although collagen deposition was noticeable at an early stage of infection (4 dpi), a significantly higher amount of collagen deposition was found at 16 dpi in SARS-CoV-2 infected, untreated hamster lungs (**Figure 4A**). Treatment with CC-11050 significantly reduced the interstitial collagen deposition (p=0.0114), compared to the untreated hamster lungs at 16 dpi (**Figure 4A** and **Supplementary Figure 2**). Consistent with these observations, the hydroxyproline content of untreated hamster lungs significantly increased between 4 to 16 dpi (p=0.0004). However, compared to the untreated infected hamsters, treatment with CC-11050 significantly reduced the lung hydroxyproline content at 16 dpi (p=0.0218) (**Figure 4B**). These results strongly suggest that CC-11050 treatment can effectively reduce lung fibrotic remodeling caused by SARS-CoV-2 infection.

**Table 1:** Lung fibrosis score.

**Treatment group (days post infection)**	**Thickening of alveolar basement membrane (0-5)**	**Interstitial collagen fibers deposition (0-5)**	**Perivascular adventitial layer proliferation (0-5)**	**Peribronchiolar fibroelastic layer proliferation** **(0-5)**	**Pleural membrane thickening (0-5)**
Untreated (4 dpi)	2	2	3	3	1
	1	0	2	0	1
	1	2.5	2.5	1	1
CC-11050 (4 dpi)	0	0.5	3.5	3	0
	1	2	2	1.5	1
	1	2	2.5	2	1
	1.5	2	3	3	1.5
Untreated (16 dpi)	2	4	3	2	2
	1	4	2	1	1.5
	2	4.5	3	3	2
CC-11050 (16 dpi)	0	1	3	2	1
	1.5	1.5	2.5	2.5	2
	1.5	1.5	3	3	2
	1.5	1.5	2.5	2	1

### CC-11050 treatment downregulated inflammatory responses in the lungs

**Figure 5:**
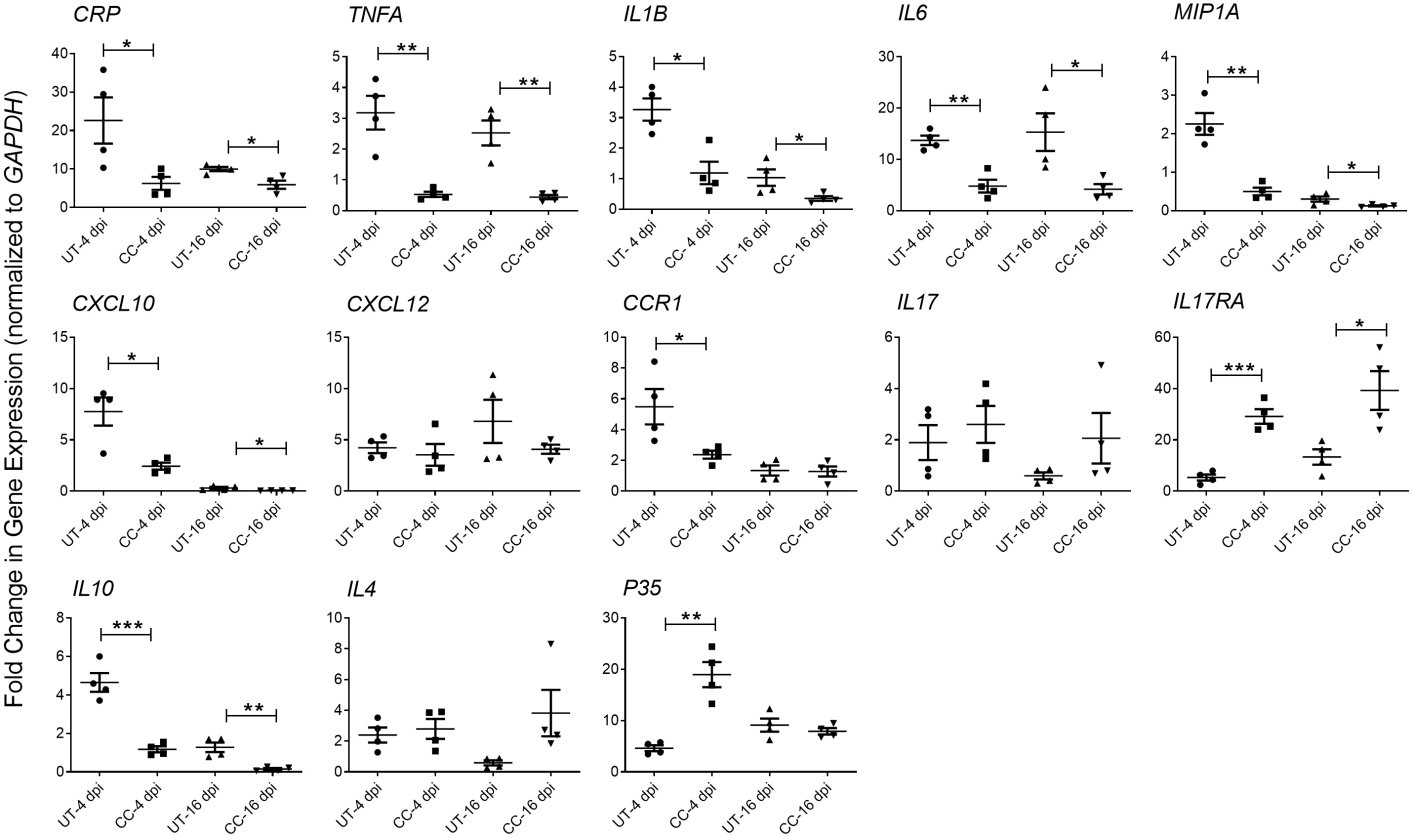
CC-11050 treatment dampens inflammatory response gene expression in SARS-CoV- 2 infected hamster lungs. Expression of *CRP*, *TNFA*, *IL1B*, *IL6*, *MIP1A*, *IL17*, *IL17RA*, *CXCL10*, *CXCL12*, *CCR1*, *IL10*, *IL4* and *P35* genes was determined in untreated (UT) or CC-11050 treated (CC), SARS-CoV-2 infected hamster lungs at 4 and 16 dpi. The expression level of each target gene was normalized to *GAPDH* levels in the same sample. The experiment was performed with four biological replicates (n=4 animals per group per time point) and repeated at least twice (technical replicates). Values plotted were mean ± SE of each group and represented as fold change in expression relative to *GAPDH* levels. Data were analyzed by the unpaired t-test. * p<0.05; **p<0.01; ***p<0.005.

**Figure 6:**
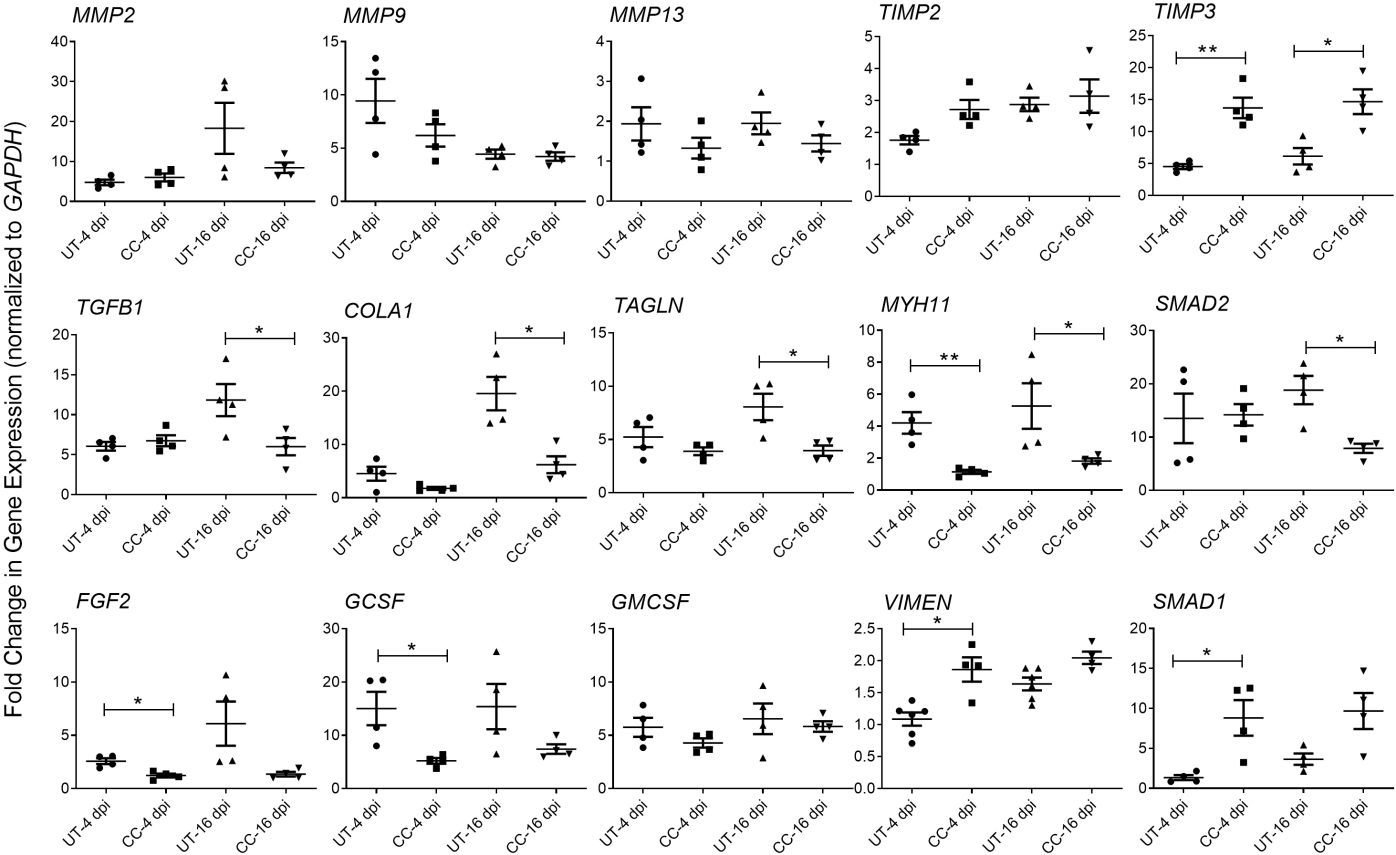
CC-11050 treatment modulates expression of genes associated with tissue remodeling and fibrosis in SARS-CoV-2 infected hamster lungs. Fold change in the expression of tissue remodeling and fibrosis genes *MMP2*, *MMP9*, *MMP13*, *TIMP2*, *TIMP3*, *TGFB1*, *COLA1*, *TAGLN*, *MYH11* and *SMAD2*, and growth factors *GMCSF*, *FGF2*, *VIMEN*, *SMAD1* and *GCSF* at 4 and 16 dpi in untreated (UT) or CC-11050 treated (CC) hamster lungs. The expression level of each target gene was normalized to *GAPDH* levels in the same sample. The experiment was performed with four biological replicates (n=4 animals per group per time point) and repeated at least twice. Values plotted were mean ± SE of each group and represented as fold change in expression relative to *GAPDH* levels. Data were analyzed by the unpaired t-test. *p<0.05; **p<0.01.

Since the cytokine storm and ARDS are associated with the severity of inflammation and disease in patients with COVID-19, we studied the effect of CC-11050 treatment on the expression of pro-inflammatory cytokines in the lungs of SARS-CoV-2 infected hamsters during early (4 dpi) and late (16-dpi) stages (**Figure 5**). As expected, SARS-CoV-2 infection upregulated the expression of the tested proinflammatory (*CRP*, *TNFA*, *IL1B*, *IL6*, *CXCL10*, *CXCL12*, *CCR1* and *MIP1A*) and anti-inflammatory (*IL10*) cytokine and chemokine genes at 4 dpi. Although the expression of several of these markers was downregulated in the untreated hamster lungs at 16 dpi, the difference was not statistically significant between 4 and 16 dpi. In contrast to the untreated animals, CC-11050 treatment significantly downregulated the expression of pro-inflammatory marker genes, including *CRP* (4dpi, p=0.0392; 16dpi, p=0.0146), *TNFA* (4dpi, p=0.003; 16dpi, p=0.0023), *IL1B* (4dpi, p=0.0072; 16dpi, p=0.0512), *IL6* (4dpi, p=0.0012; 16dpi, p=0.0265), *MIP1A* (4dpi, p=0.0011; 16dpi, p=0.0434), *CXCL10* (4dpi, p=0.0093; 16dpi, p=0.0482) and *CCR1* (4dpi, p=0.0382; 16dpi, p=ns), whereas expression of *IL17RA* (4dpi, p=0.0002; 16dpi, p=0.019), and *p35* (4dpi, p-0,0012) was significantly increased (**Figure 6**). Together, the expression patterns of selected “cytokine storm” markers in the hamster lungs indicate that CC-11050 treatment alleviate the exacerbated inflammation caused by SARS-CoV-2 infection.

### CC-11050 treatment downregulated the expression of markers associated with tissue damage and fibrosis in the lungs

Tissue fibrosis is an important consequence of inflammation and ARDS during SARS-CoV-2 infection (29–31). The RNAseq analysis of SARS-CoV-2 infected hamster lungs at 4 and 16 dpi, showed a significant upregulation of the expression of genes involved in the lung fibrosis network (**Supplementary Figure 3**). Of the 38 differentially expressed genes in this network, 16 genes were upregulated, 13 genes were downregulated, and 9 genes were not significantly expressed in the SARS-CoV-2 infected hamster lungs, compared to the uninfected controls at 4 dpi. By 16 dpi, 20 genes were upregulated and 18 downregulated, suggesting an increase in the activation of lung fibrosis at a later stage of SARS-CoV-2 infection. To determine whether CC-11050 treatment exerts an anti-fibrotic effect in SARS-CoV-2 infected hamster lungs, we analyzed the expression of genes that play essential roles in fibrotic remodeling in the lung. Specifically, the level of *MMP2*, *MMP9*, *MMP13*, *TIMP3*, *TGFB1*, *COL1A1*, *TAGLN*, *MYH1* and *SMAD2* expression was quantified by real-time PCR at 4 and 16 dpi. Compared to the untreated SARS-CoV-2 infected hamster lungs, in CC-11050 treated animals downregulated the expression of *MMP2*, *MMP9* and *MMP13*, although the difference was not statistically significant. In contrast, the expression of *TIMP3*, a negative regulator of MMPs, was significantly upregulated in CC-11050 treated animals at 4 dpi (p=0.0014) and 16 dpi (p=0.0105) (**Figure 6**). In addition, CC-11050 treatment significantly downregulated the expression of genes that play a key role in lung fibrosis, such as *TGFB1* (16 dpi; p=0.0433), *COL1A1* (16 dpi, p=0.009), *TAGLN* (16 dpi; p=0.0218), *MYH11* (4 dpi; p=0.0042; 16 dpi; p=0.05) and *SMAD2* (16 dpi; p=0.008) (**Figure 6A**). Furthermore, the SARS-CoV-2 infection-induced expression of cellular growth factors (*FGF2*, and *GCSF*) in the hamster lungs, was significantly downregulated upon CC-11050 treatment [*FGF2* (4 dpi, p=0.0067) and *GCSF* (4 dpi, p=0.0214)] (**Figure 6B**).

**Figure 7:**
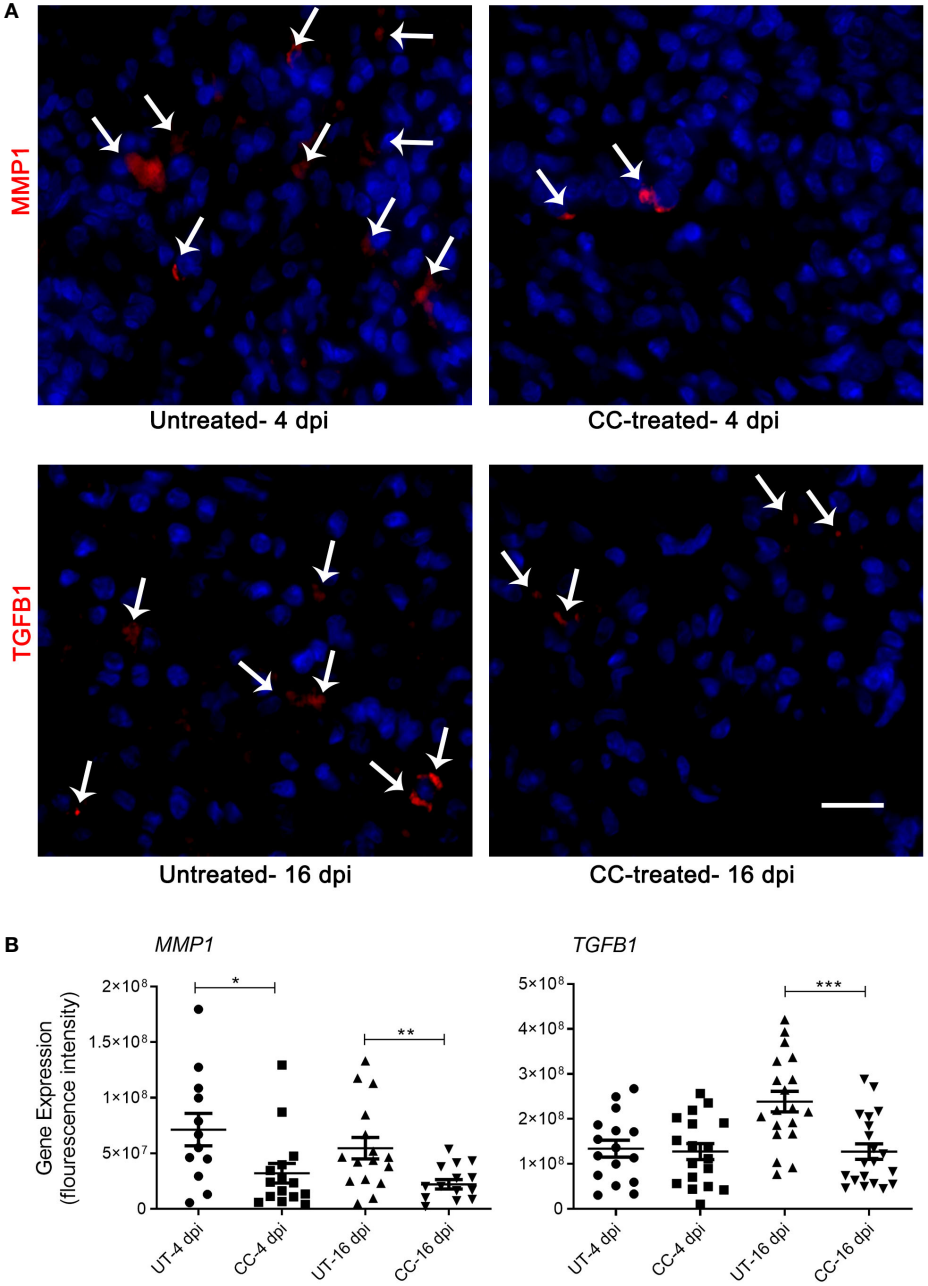
Spatial expression of MMP1 and TGFB1 in SARS-CoV-2 infected hamster lungs. **(A)** Representative image of *MMP1* and *TGFB1* expression determined by smRNA-FISH method using lung sections of SARS-CoV-2 infected hamsters with or without CC-11050 treatment at 4 and 16 dpi. White arrows indicate the cells positive for *MMP1* and *TGFB1* (red color spots); DAPI stain was used to visualize the cell nucleus (blue color). Images were taken in 630x magnification from at least 10 fields per slide/sample (n=3 animals per group per time point). The scale bar represents 50μm. **(B)** The mean fluorescent signal intensities of target gene transcripts (*MMP1* and *TGFB1*) were measured and corrected for background levels using ImageJ software. Average signal intensity data from at least 10 fields per slide/sample (n=3 animals per group per time point) were plotted as mean ± SE. UT-untreated; CC-CC-11050 treated. Statistical analysis was carried out using the unpaired t-test. * p<0.05; **p<0.01; ***p<0.005.

The spatial expression of MMP-1 and TGF- β genes was quantified in the lungs by the smRNA-FISH method. An increased expression of *MMP1* was found in untreated infected hamsters at both 4 and 16 dpi. Compared to the untreated hamsters, the CC-11050 treated hamsters showed significantly reduced expression of *MMP1* in the lungs at 4 dpi (p=0.0245) and 16 dpi (p=0.0055) (**Figure 7**). An increased expression of *TGFB1* was observed in the untreated hamster lungs at 16 dpi, compared to 4 dpi. In contrast, administration of CC-11050 significantly downregulated *TGFB1* expression in the lungs, compared to untreated animals at 16 dpi (p=0.0004) (**Figure 7**).

### CC-11050 treatment decreased the production of TNF-α, IL-6 and TGF-β in the lungs

**Figure 8:**
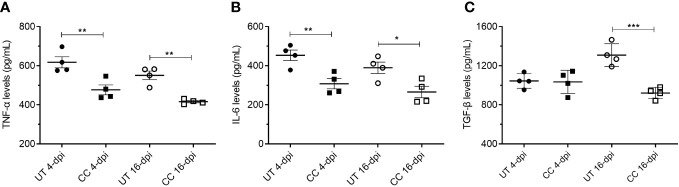
CC-11050 treatment significantly reduced TNF-α, IL-6 and TGF-β levels in the SARS-CoV-2 infected hamster lungs. TNF-α **(A)**, IL-6 **(B)** and TGF-β **(C)** levels in the lung homogenates at 4-dpi and 16-dpi were determined by ELISA and the amount of cytokine produced was presented as pg/mL of lung homogenates. Cytokines levels were determined from four biological replicates (n=4 animals per group per time point) in duplicates. Values were plotted as mean ± SE of each group. UT-untreated; CC-CC-11050 treated. Data were analyzed by the unpaired t-test. * p<0.05; **p<0.01, ***p<0.005.

In addition to the mRNA expression, we also estimated the protein levels of key cytokines such as TNF-α, IL-6 and TGF-β in the lung homogenates (4 and 16 dpi), which play key roles in inflammation, tissue remodeling and fibrosis. Similar to mRNA expression profile, administration of CC-11050 significantly downregulated the levels of TNF-α (4 dpi: p=0.0099; 16 dpi: p=0.0011), IL-6 (4 dpi: p=0.0085; 16 dpi: p=0.0233) and TGF-β (16 dpi: p=0.0010) (**Figure 8**). These results suggest that CC-11050 treatment significantly dampens exacerbated inflammation and lung fibrotic remodeling during and after recovery from disease.

### Administration of CC-11050 reduced the infiltration of immune cells and reticular fibroblasts into the lungs

**Figure 9:**
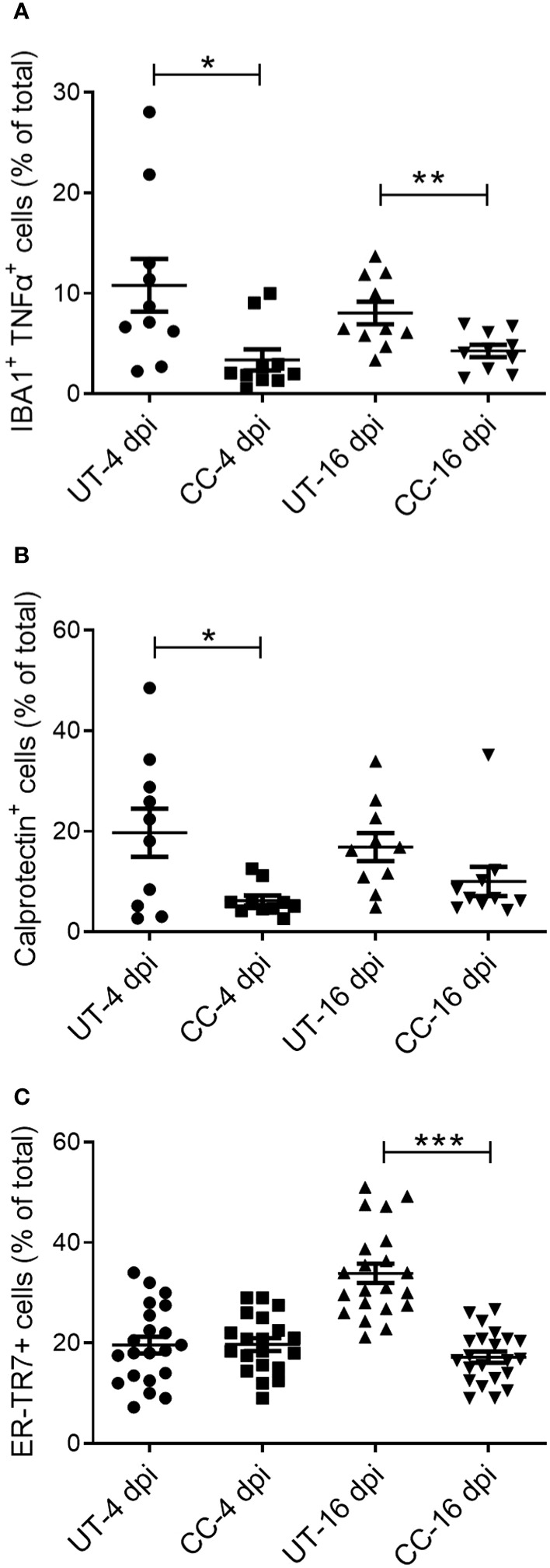
CC-11050 treatment decreases the frequency of activated macrophages, granulocytes, and reticular fibroblasts in SARS-CoV-2 infected hamster lungs. Expression of cell-specific markers was determined by immunohistochemistry of lung sections using respective antibodies. **(A)** The percentage of activated macrophages (IBA1+/TNF-α+) in untreated (UT) and CC-11050 treated (CC) hamster lungs at 4- and 16-days post SARS-CoV-2 infection. **(B)** The percentage of activated neutrophils (Calprotectin+) in untreated (UT) and CC-11050 treated (CC) hamster lungs at 4- and 16-days post SARS-CoV-2 infection. **(C)** The percentage of activated reticular fibroblasts (ER-TR7+) in untreated (UT) and CC-11050 treated (CC) hamster lungs at 4- and 16- days post SARS-CoV-2 infection. The total number of cells and those positive for a specific marker (based on mean fluorescent intensity) were enumerated manually in each field analyzed. At least 10 fields per slide/sample (n=3 animals per group per timepoint) were measured and analyzed using ImageJ software. Statistical analysis was carried out using the unpaired t-test. Values plotted were mean ± SE. * p<0.05; **p<0.01; ***p<0.005.

Activation of immune cells is a key factor that contributes to the exacerbated inflammation and fibrosis in the lungs of COVID-19 cases (2–4). Therefore, we evaluated the spatial distribution of activated macrophages (IBA1+), neutrophils (Calprotectin+), CD4+ T cells, and reticular fibroblasts (ER-TR7+) in SARS-CoV-2 infected hamster lungs at 4 and 16 dpi by immunohistochemistry followed by quantitative analysis (**Figure 8** and (**Supplementary Figure 4**). An increased number of activated macrophages producing TNF-α (IBA1+ TNF-α+ cells) was observed in the lungs during the acute stage of infection (4 dpi), and to a lesser extent during recovery from the infection (16 dpi) (**Figures 9A, B**). Compared to the untreated group, CC-11050 treatment significantly decreased the frequency of IBA1+ TNF-α+ activated macrophages at 4 dpi (p=0.0172) and 16 dpi (p=0.0083) (**Figure 9B**). Similarly, an increased neutrophil (Calprotectin+ cells) accumulation was observed at 4 dpi, compared to 16 dpi, in the untreated hamster lungs (**Figures 9C, D**). CC-11050 treatment significantly reduced the percentage of the activated neutrophils in the lungs, compared to the untreated hamsters at 4 dpi (p=0.0132) (**Figure 9D**). In addition, we determined the frequency of reticular-fibroblast (ER-TR7+ cells) in the lungs, which play an important role in wound healing and fibrosis. An increased frequency of ER-TR7+ cells was observed in the untreated hamsters at 16 dpi, compared to 4 dpi (**Figure 9E**). The number of ER-TR7+ reticular fibroblasts was significantly decreased in the lungs of CC-11050 treated hamsters at 16 dpi (p<0.0001), compared to the untreated animals (**Figure 9F**). No significant difference in the distribution of CD4+ cells was noted in the lungs of untreated versus CC-11050 treated hamsters at 4 or 16 dpi (data not shown). Together, these findings suggest that the anti-inflammatory and anti-fibrotic activities of CC-11050 in SARS-CoV-2 infected hamster lungs are associated with reduced accumulation of activated macrophages, neutrophils, and reticular fibroblasts in the lungs.

### CC-11050-treatment downmodulates fibroblast activation and differentiation

**Figure 10:**
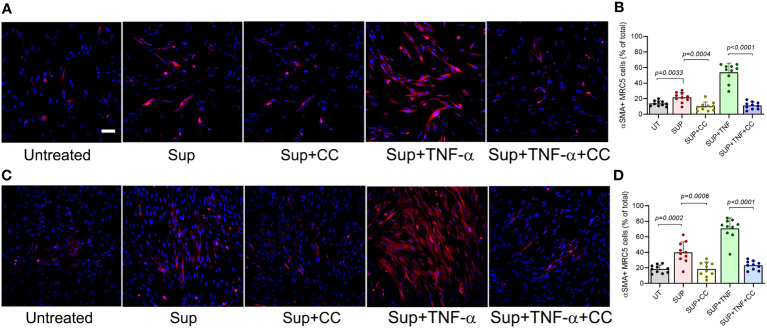
CC-11050 treatment downregulates the production of αSMA protein in human lung fibroblasts. **(A)** Representative immunofluorescence images of MRC-5 cells treated without (Untreated) or with the culture supernatant from SARS-CoV2-infected Vero E6 cells (Sup) and treated with CC-11050 (CC), TNF-α, or TNF-α plus CC-11050 (TNF-α+CC) for 24 hours **(A, B)** or 48 hours **(C, D)** post-treatment. At specific time points, cells were fixed, permeabilized, blocked, and stained with anti-αSMA primary antibody and Texas Red conjugated secondary antibody, and nuclei were stained with DAPI dye. Images were taken at 200x magnification. The percentage of αSMA+ cells was determined by counting the positive cells from 10 randomly acquired fields (n=3 animals per group per timepoint) using ImageJ software. Statistical analysis was carried out using the unpaired t-test. Values plotted were mean ± SE.

Our analysis showed that CC-11050 treatment significantly downregulated the expression TNF-α and recruitment of TNF-α + macrophages to the lungs, with a concomitant decrease in lung fibrosis and fibroblast cell activation in the SARS-CoV2 infected hamsters. It has been demonstrated that TNF-α stimulates human fibroblasts and promotes fibrocyte differentiation in the lungs of pulmonary fibrosis patients and *in vitro* models of fibrosis (19–21). Therefore, we hypothesized that TNF-α is a regulator of fibroblast activation, fibrotic remodeling and tissue fibrosis. We further hypothesized that CC-11050 dampens fibrotic remodeling through its ability to downregulate TNF-α production and associated inflammation. To test these hypotheses, we first determined the TNF-α mediated fibroblast differentiation into myofibroblast, by determining the production of alpha-smooth muscle actin (αSMA). Since human lung fibroblast cells (MRC-5) are refractory to SARS-CoV-2 infection, we treated these cells with the conditioned medium (Sup) from SARS-CoV2-infected Vero-6 Cells and cultured them in the presence or absence of TNF-α and/or CC-11050. The percentage of αSMA+ cells was determined at 24 and 48 hr post-treatment (**Figure 10**).

Compared to the untreated control cells, a significantly increased fraction of αSMA+ cells were noted in the Sup-treated fibroblasts at 24hr (p=0.003) and 48hr (p=0.0002) post-treatment (**Figure 9**). The percentage of αSMA+ cells further increased when the Sup-treated cells were also treated with TNF-α. In contrast, the addition of CC-11050 to Sup-treated cells significantly reduced the frequency of αSMA+ cells at 24hr (p=0.0004) and 48hr (p=0.0006) post-treatment, compared to the untreated control cells (**Figure 10**). In addition, among the infected cells, CC-11050 treatment significantly reduced the frequency of αSMA+ fibroblasts, compared to those treated with TNF-α at 24 hr (p<0.0001) and 48 hr (p<0.0001) post-treatment. These observations indicate that (i). Conditioned media of SARS-CoV-2 infected Vero-E6 cells can induce fibroblast activation and differentiation into myofibroblast, which is further augmented by TNF-α treatment. (ii). The activation and differentiation of fibroblasts by conditioned media and TNF-α were dampened significantly by CC-11050 treatment.

## Discussion

In this study, we show that treatment with the immune modulatory PDE4i drug CC-11050 accelerates the reduction in the replicative viral titer at 4 dpi and alleviates pulmonary disease pathology in a hamster model of SARS-CoV-2 infection. However, CC-11050 did not have direct antiviral activities when incubated with SARS-CoV-2. Therefore, the decrease in lung viral titer of CC-11050 treated hamster as compared to the untreated hamster at 4 dpi is attributed to the host immune-mediated control of viral replication. CC-11050, a host phosphodiesterase-4 inhibitor, dampens the production of TNF-α, a pro-inflammatory cytokine and modulates subsequent downstream responses. Further, inhibition of PDE4 results in the accumulation of intracellular cyclic AMP, an anti-inflammatory signaling molecule. Consistently, during SARS-CoV-2 infection, CC-11050 treatment dampened the expression of the inflammatory “cytokine storm” markers and reduced the recruitment of inflammatory cells into the lungs. Furthermore, CC-11050 therapy reduced the extent of lung fibrotic remodeling and associated gene expression. Finally, we showed that the CC-11050-mediated reduction of lung fibrotic remodeling was associated with the drug’s ability to dampen TNF-α production. Together, these observations suggest that the combined effect of TNF-α reduction and cAMP accumulation enables the host cells to control SARS-CoV-2 replication and infection effectively, without triggering inflammation, which would contribute to exacerbated host cell death and disease pathology. These results are consistent with and extend our previous reports in the mouse and rabbit models of pulmonary TB, where adjunctive CC-11050 treatment significantly accelerated the antibiotic killing of *Mycobacterium tuberculosis* in the lungs, and reduced lung tissue fibrosis, compared to treatment with antibiotics alone (10, 16).

SARS-CoV-2 infection triggers extensive production of pro-inflammatory molecules such as CRP, TNF-α, IL-1β, IL-6, MIP-1α, IP-10 and IL-10 by activated inflammatory monocytes and neutrophils recruited into the lungs of COVID-19 patients (32–34). The elevated production of pro-inflammatory cytokines contributes to the inflammatory “cytokine storm”, exacerbating disease pathology in the lungs of patients with ARDS, a serious immunopathology of COVID-19 (32, 33, 35, 36). We observed an increased frequency of activated macrophages (TNF-α+), neutrophils, and reticular fibroblasts in the lungs of SARS-CoV-2-infected hamsters. Elevated TNF-α production by activated macrophages can potentiate fibroblast differentiation and promote collagen accumulation in myofibroblasts, while increased neutrophil influx mediates chemotaxis of inflammatory macrophages to the site of infection and plays a significant role in inflammation, ARDS, and fibrosis in the lungs (22, 37, 38). Previous studies have reported that dysregulated neutrophil infiltration and subsequent formation of neutrophil extracellular trap (NET) contribute to the pathophysiology of inflammation, organ damage, and immuno-thrombosis in COVID-19 cases (39, 40). In addition, the ER-TR7+ fibroblasts secrete an extracellular matrix that contributes to collagen type VI deposition, as well as chemokines that mediate the recruitment of T cells, B cells, and dendritic cells from the blood and lymph flow (41, 42). The recruitment pattern of these key cells and markers of tissue inflammation and fibrosis during SARS-CoV-2 infection of hamster lungs is consistent with the corresponding findings in patients with COVID-19. Importantly, the administration of CC-11050 dampened TNF-α expression and downregulated macrophage activation and neutrophil recruitment, all of which may contribute to the control of immunopathology and tissue damage in the lungs. Results from a clinical trial that tested the beneficial effects of CC-11050 treatment in patients with cavitary TB clearly demonstrated significant improvement in lung function (9). Similarly, an earlier study has shown that treatment with a PDE4i such as roflumilast and rolipram controlled the neutrophil recruitment by reducing TNF-α, IL-6, CXCL1, and CXCL2/3 in a rabbit model of pulmonary TB (17).

We also observed a significantly decreased frequency of ER-TR7+ reticular fibroblast in the lungs of CC-11050 treated infected hamsters. Lung fibrosis is one of the most important clinical complications of COVID-19 that develops primarily following tissue damage caused by excessive inflammation driven by viral antigen, drug-induced toxicity, and hyperoxia-induced acute lung injury, secondary to mechanical ventilation (43, 44). Moreover, patients who recovered from COVID-19 (Long-COVID) can suffer from long-term immunopathogenesis related to ARDS, including irreversible interstitial lung disease and fibrosis that contribute to early mortality. Several studies reported that 30% to 50% of COVID-19 survivors have post-COVID-19 lung fibrosis and patients with severe COVID-19 pneumonia were at high risk of sustained lung fibrosis seen within three months of follow-up (44–46). Consistent with these clinical findings, we observed an excessive collagen deposition in the lungs of SARS-CoV-2 infected untreated hamsters that was prominent during the disease recovery phase (i.e., 16 dpi).

The onset of fibrosis in different organs, including the lungs, is regulated by TGF-β, and the SMAD-dependent TGF-β signaling pathways are key therapeutic targets for the management of fibrosis (47). The expression of several markers of fibrosis and lung remodeling such as TGF-β1, TAGLN, MYH11, SMAD2, and MMPs and growth factors such as GCSF and FGF, is increased during SARS-CoV2 infection in our hamster model. Importantly, CC-11050 treatment significantly decreased the accumulation of collagen fibers and TGF-β expression in the lungs of these animals. Mechanistically, PDE4 inhibition leads to elevated levels of cAMP and increased CREB phosphorylation and activated CREB competitively binds to the CREB-binding protein (CBP), which blocks TGF-β dependent SMAD/CBP binding (48–50). Our observations were in-line with an earlier study that showed PDE4i exert anti-fibrotic effects by inhibiting TGF-β1 and SMAD-dependent signaling pathways in lung fibroblasts (23).

Host-directed therapy (HDT) is emerging as a new treatment approach for infectious diseases, where host immune and/or cellular responses are modulated through the administration of a small molecule to improve the clinical outcome of treatment (51, 52). Several HDT agents that modulate immune responses such as antimicrobial peptide synthesis, autophagy induction, inflammation, and cell-mediated immunity and immunological memory have shown promising results for a potential adjunct treatment option for infectious diseases including TB and COVID-19 (53). Accordingly, several clinical strategies, such as administration of corticosteroids (e.g. methylprednisolone, dexamethasone), tyrosine kinase inhibitors (e.g. baricitinib, imatinib), and cytokine inhibitors (e.g. infliximab and tocilizumab) have been investigated to control inflammation and immunopathology caused by SARS-CoV-2 infection (2, 52). These steroid and non-steroid anti-inflammatory agents have been tested in patients with severe COVID-19 symptoms, such as ARDS, to curb the exacerbated inflammation and to improve lung function (52–56). In clinical studies, dexamethasone therapy showed a 35% reduction in the mortality rate of severe COVID-19 cases admitted to intensive care units (57, 58).

However, corticosteroid therapy has been implicated in immunosuppression, inducing lymphopenia in patients with cancer and diabetes. Extended periods of corticosteroid therapy are associated with health risks, including hormonal imbalance, weight reduction, myalgia, vomiting and arthralgia (59, 60). Similarly, anti-IL-6 and anti-IL-6R antibodies, such as Sarilumab, Siltuximab and Tocilizumab have been tested for their ability to control virus-induced hyper-inflammation in COVID-19 patients (61–63). In clinical trials, these therapeutic agents showed mixed responses in improving the clinical status of patients with COVID-19.64-66 For example, Tocilizumab therapy extended the survival of severe COVID-19 cases with pneumonia and moderate-to-severe hypoxia (PaO2/FiO2 ratio <150) and improved the viral clearance (64, 65). However, this drug did not show significant therapeutic value in patients with mild hypoxia (PaO2/FiO2 ratio of 200-300) compared to the standard supportive care (66). Furthermore, the side effects of anti-IL-6 and anti-IL-6R antibody therapy include neutropenia, abnormal liver function, and respiratory infections, including TB (63).

Other studies have shown that the administration of infliximab (anti-TNF-α antibody) reduced IL-6 and CRP levels in COVID-19 cases (67, 68). However, infliximab therapy did not significantly improve the mortality risk of severe COVID-19 cases, although it helped to reduce the length of hospital stay of these patients (67, 68). Furthermore, anti-TNF-α antibody therapy is linked to side effects such as sarcoidosis, uveitis, and nervous system disorders; and immunosuppression associated with TNF-α blockade is a significant risk factor for exacerbation of disease pathology in SARS-CoV-2 infected individuals (69–71).

Since proinflammatory cytokines such as TNF-α and IL-6 are also crucial for host control of bacterial and viral infections, therapeutic interventions that selectively target these cytokines are associated with adverse side effects, including delayed pathogen clearance, hypotension, multi-organ dysfunction, and reactivation of secondary infections such as *M. tuberculosis* infection (72). In contrast, CC-11050, the PDE4i tested in this study, did not cause systemic immunosuppression of the treated host, while alleviating exacerbated inflammation, tissue destruction, and fibrosis, all of which augmented the viral clearance and contributed to improved lung function by reducing fibrosis. In addition to CC-11050, the beneficial effect of another phosphodiesterase 4B inhibitor (BI 1015550) has been tested in patients with idiopathic pulmonary fibrosis (IPF) and other forms of progressive pulmonary fibrosis (72–75). This study reported that treatment of 147 IPF patients with BI 1015550 for 12 weeks increased forced vital capacity (FVC) and improved lung function. However, in the phase three clinical trial, several patients discontinued the BI 1015550 treatment due to adverse effects such as diarrhea (72). Moreover, treatment of fibroblasts isolated from IPF patients with BI 1015550 inhibited the TGF-β-induced myofibroblast transformation, the expression of extracellular matrix protein as well as fibroblast growth factor plus interleukin-1β-induced cell proliferation (76). These studies suggest that PDE4i treatment has the potential to improve fibrotic remodeling and thus, for the better management of pulmonary fibrosis.

In conclusion, we demonstrated that administration of CC-11050 effectively suppressed severe inflammation, downregulated the recruitment of neutrophils, activated macrophages, and the frequency of reticular fibroblast, and reduced the immunopathology of disease in the lungs of SARS-CoV-2 infected hamsters thereby reducing pathogen-induced tissue damages and subsequent fibrotic remodeling. Additional studies, including the evaluation of CC-11050 therapy for long-term SARS-CoV-2 infection, are warranted to evaluate the beneficial role of CC-11050 with and without adjunctive anti-viral therapy for better management of COVID-19 and post-COVID-19 complications.

## Strengths and limitations of the study

This preclinical proof of concept report demonstrates that oral administration of a PDE4i, CC-11050, dampens inflammation and fibrotic remodeling in the lungs during SARS-CoV-2 infection. Since the focus of this study was to evaluate the immunomodulatory and therapeutic effects of CC-11050, during SARS-CoV-2 infection, we did not evaluate the adjunctive effect of CC-11050 in combination with anti-viral drugs in the hamster model. Our previous studies in animal models of TB have shown significant improvement in bacterial clearance and lung fibrosis when CC-11050 was administered together with anti-TB drugs. Based on those observations, we predict a similar host-beneficial effect in SARS-CoV-2 infected hamsters when CC-11050 is administered along with an antiviral drug. In this study, the effect of CC-11050 was evaluated using the SARS-CoV-2-WA-1/2000 strain for the infection of hamsters. However, whether the effect of CC-11050 would be impacted by the nature of the infecting virus strains, such as the Omicron or BA.5 variants, remains to be tested.

## Data availability statement

The original contributions presented in the study are included in the article/**Supplementary Material**. Further inquiries can be directed to the corresponding author.

## Ethics statement

Ethical approval was not required for the studies on humans in accordance with the local legislation and institutional requirements because only commercially available established cell lines were used. The animal study was approved by Rutgers Institutional Animal Care and Use Committee and Institutional Biosafety Committee. The study was conducted in accordance with the local legislation and institutional requirements.

## Author contributions

AK: Data curation, Writing – review & editing, Formal Analysis, Investigation, Methodology, Validation, Visualization, Writing – original draft. SR: Formal Analysis, Investigation, Methodology, Visualization, Writing – review & editing. RK: Formal Analysis, Investigation, Methodology, Visualization, Writing – review & editing, Data curation, Validation. AN: Investigation, Methodology, Writing – review & editing. GK: Writing – review & editing, Resources. SS: Resources, Writing – review & editing, Conceptualization, Data curation, Formal Analysis, Funding acquisition, Project administration, Supervision.

## Supplementary material

Supplementary Figure 1 Evaluation of direct anti-SARS-CoV-2 activities of CC-11050. Infectious, replicative SARS-CoV-2 was treated with CC-11050 at 0.5 and 5uM or left untreated for 24 hours. The viability of replicative SARS-CoV-2 was calculated by PFU assay. N=3 wells per condition repeated at least twice. Statistical analysis was carried out using One-way Anova. Values plotted are mean+/-SD. ns-not significantly different between untreated and CC-11050-treated groups.

Supplementary Figure 2 Low magnification images of lung fibrotic remodeling. Lung sections from SARS-CoV-2 infected hamsters at 4 dpi and 16 dpi with or without CC-11050 treatment were stained by Masson’s trichrome method. The collagen deposition is visible as blue coloration. n=4 per group/timepoint. Image photographed at 40x magnification. Scale bar refers to 200 µm, applicable to all images.

Supplementary Figure 3 Network analysis and heatmap of genes involved in fibrosis and tissue remodeling in hamster lungs. Significantly differentially expressed genes (SDEG) in hamster lungs infected with SARS-CoV-2 at 4 and 16 dpi, compared to uninfected controls were derived from RNAseq data. Ingenuity Pathway Analysis (IPA) was used to analyze SDEGs. **(A)** Network analysis showing interacting partner genes involved in tissue remodeling and fibrosis in SARS-CoV-2 infected hamster lungs at 16 dpi. Green color denotes downregulation and red color denotes upregulation of genes. Solid lines indicate direct interactions and broken lines indicate indirect interactions between network genes. **(B)** Heat map of SDEGs involved in tissue remodeling and fibrosis in SARS-CoV-2 infected hamster lungs at 4 and 16 dpi. Red color indicates upregulation and blue color indicates downregulation of SDEG. The scale bar ranges from +5 (red) to -5 (blue) fold change in SDEG expression. N=3 animals per group per time point.

Supplementary Figure 4 Spatial images of SARS-CoV-2 infected hamster lungs with or without CC-11050 treatment. Representative images showing expression of immune cell-specific markers in hamster lung sections at 4 and 16 dpi. **(A)** The distribution of activated macrophages (IBA1+/TNF+) in untreated (UT) and CC-11050 treated (CC) hamster lungs at 4 and 16 days post SARS-CoV-2 infection. **(B)** The distribution of activated neutrophils (Calprotectin+) in untreated (UT) and CC-11050 treated (CC) hamster lungs at 4 and 16 days post SARS-CoV-2 infection. **(C)** The distribution of activated reticular fibroblasts (ER-TR7+) in untreated (UT) and CC-11050 treated (CC) hamster lungs at 4- and 16- days post SARS-CoV-2 infection. Representative image from at least 10 fields per slide/sample (n=3 animals per group per timepoint) was shown. The scale bar represents 50μm, applicable to all images. Quantitative data of the image analysis is presented in.
