# Supplementary figures and images for: A phosphodiesterase-4 inhibitor reduces lung inflammation and fibrosis in a hamster model of SARS-CoV-2 infection
Source: Front Immunol. 2023 Oct 2;14:1270414. doi: 10.3389/fimmu.2023.1270414 (PMC10580809; doi:10.3389/fimmu.2023.1270414)

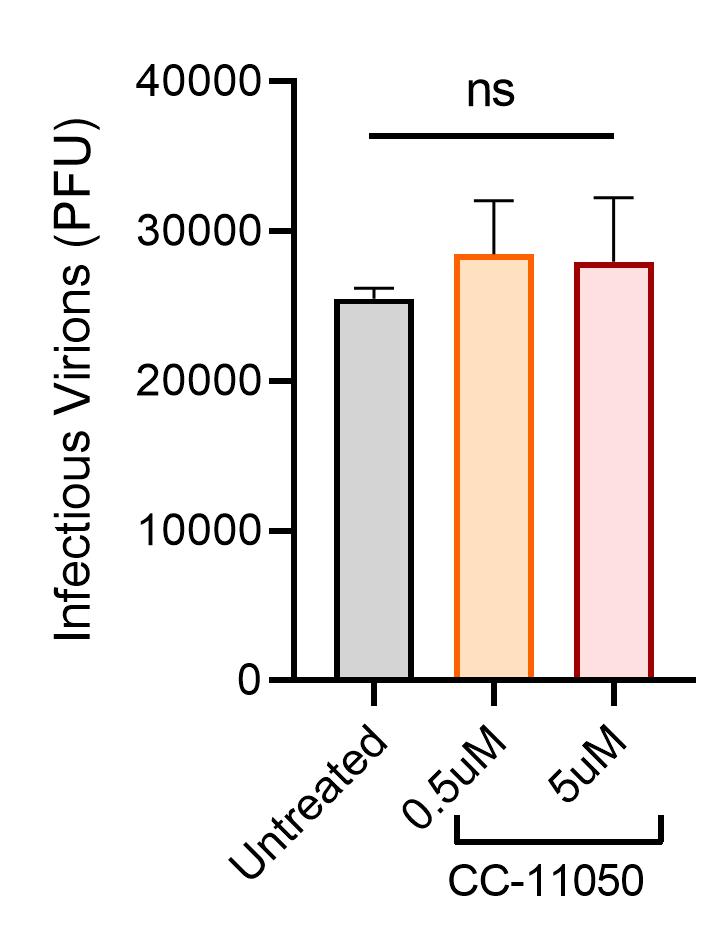

Supplement: Supplementary Figure 1 — Evaluation of direct anti-SARS-CoV-2 activities of CC-11050. Infectious, replicative SARS-CoV-2 was treated with CC-11050 at 0.5 and 5uM or left untreated for 24 hours. The viability of replicative SARS-CoV-2 was calculated by PFU assay. N=3 wells per condition repeated at least twice. Statistical analysis was carried out using One-way Anova. Values plotted are mean+/-SD. ns-not significantly different between untreated and CC-11050-treated groups. [file Image_1.jpeg]

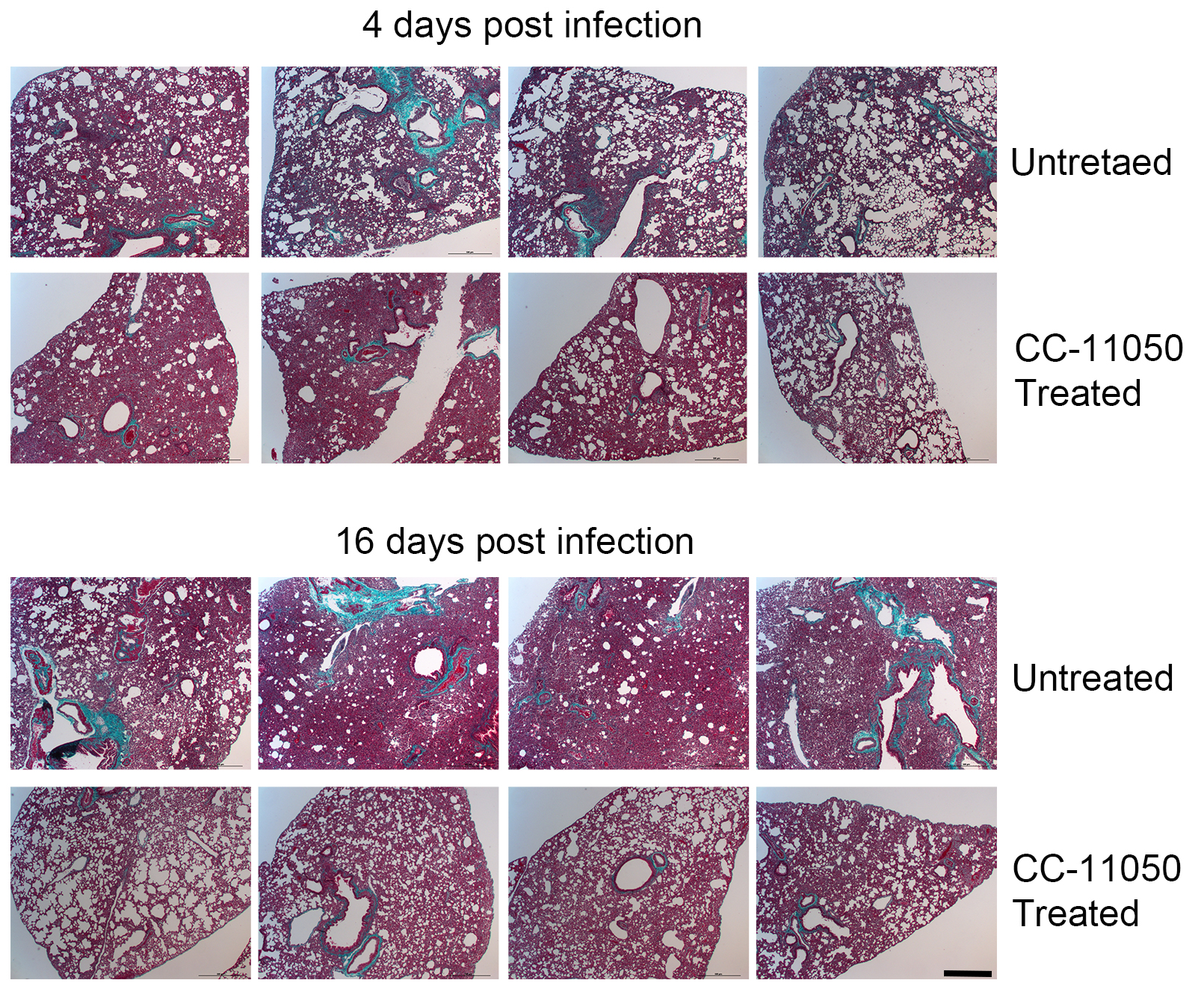

Supplement: Supplementary Figure 2 — Low magnification images of lung fibrotic remodeling. Lung sections from SARS-CoV-2 infected hamsters at 4 dpi and 16 dpi with or without CC-11050 treatment were stained by Masson’s trichrome method. The collagen deposition is visible as blue coloration. n=4 per group/timepoint. Image photographed at 40x magnification. Scale bar refers to 200 µm, applicable to all images. [file Image_2.jpeg]

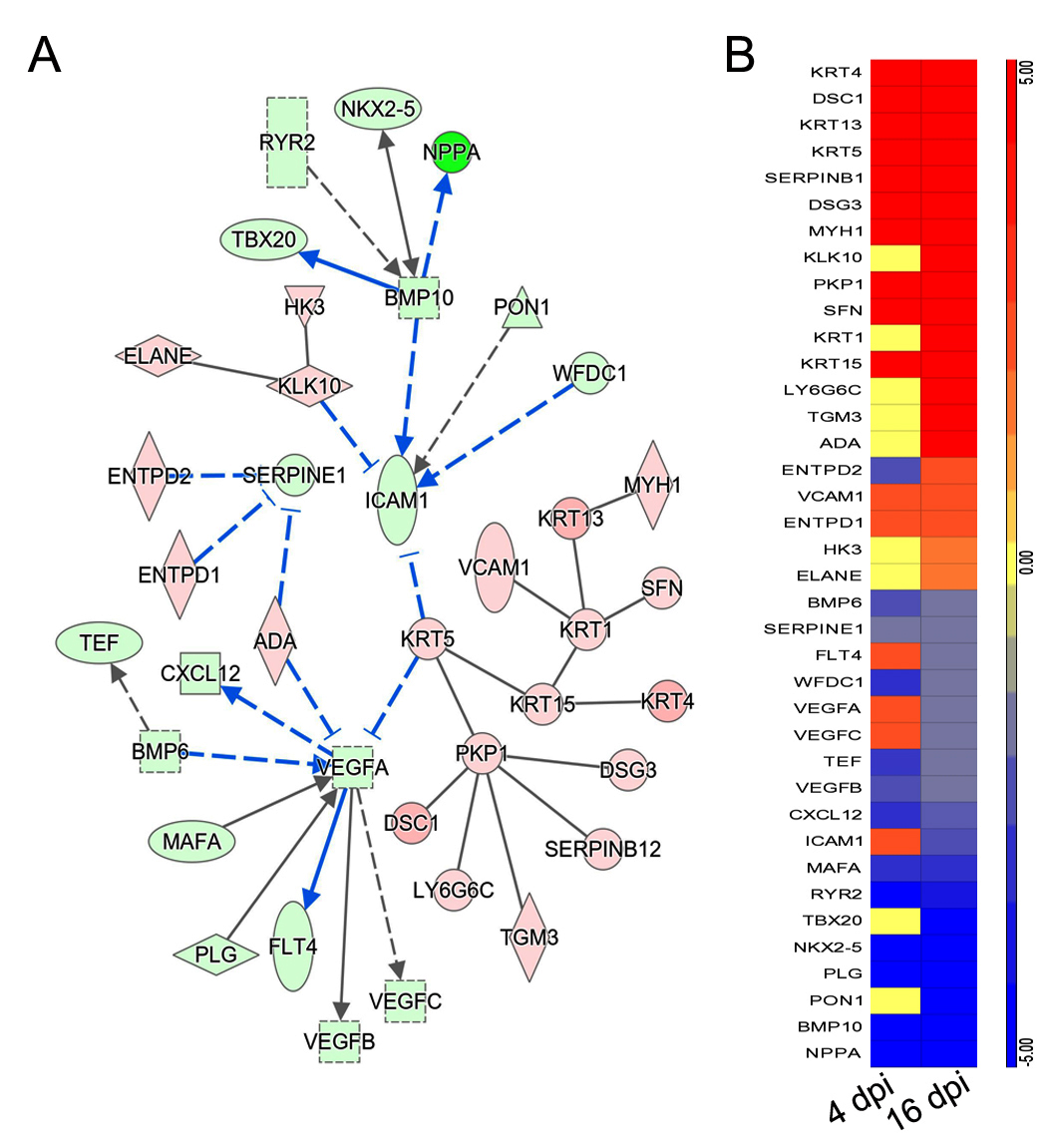

Supplement: Supplementary Figure 3 — Network analysis and heatmap of genes involved in fibrosis and tissue remodeling in hamster lungs. Significantly differentially expressed genes (SDEG) in hamster lungs infected with SARS-CoV-2 at 4 and 16 dpi, compared to uninfected controls were derived from RNAseq data. Ingenuity Pathway Analysis (IPA) was used to analyze SDEGs. (A) Network analysis showing interacting partner genes involved in tissue remodeling and fibrosis in SARS-CoV-2 infected hamster lungs at 16 dpi. Green color denotes downregulation and red color denotes upregulation of genes. Solid lines indicate direct interactions and broken lines indicate indirect interactions between network genes. (B) Heat map of SDEGs involved in tissue remodeling and fibrosis in SARS-CoV-2 infected hamster lungs at 4 and 16 dpi. Red color indicates upregulation and blue color indicates downregulation of SDEG. The scale bar ranges from +5 (red) to -5 (blue) fold change in SDEG expression. N=3 animals per group per time point. [file Image_3.jpeg]

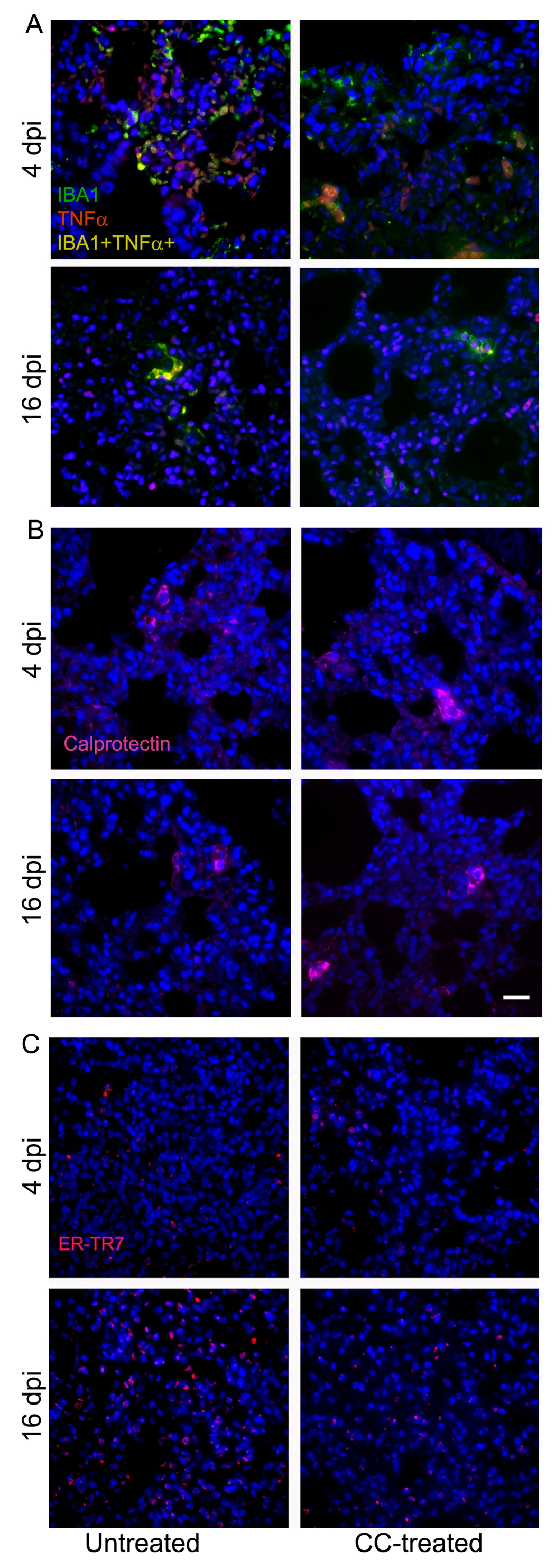

Supplement: Supplementary Figure 4 — Spatial images of SARS-CoV-2 infected hamster lungs with or without CC-11050 treatment. Representative images showing expression of immune cell-specific markers in hamster lung sections at 4 and 16 dpi. (A) The distribution of activated macrophages (IBA1+/TNF+) in untreated (UT) and CC-11050 treated (CC) hamster lungs at 4 and 16 days post SARS-CoV-2 infection. (B) The distribution of activated neutrophils (Calprotectin+) in untreated (UT) and CC-11050 treated (CC) hamster lungs at 4 and 16 days post SARS-CoV-2 infection. (C) The distribution of activated reticular fibroblasts (ER-TR7+) in untreated (UT) and CC-11050 treated (CC) hamster lungs at 4- and 16- days post SARS-CoV-2 infection. Representative image from at least 10 fields per slide/sample (n=3 animals per group per timepoint) was shown. The scale bar represents 50μm, applicable to all images. Quantitative data of the image analysis is presented in. [file Image_4.jpeg]
