# Supplementary material for: A phosphodiesterase-4 inhibitor reduces lung inflammation and fibrosis in a hamster model of SARS-CoV-2 infection
Source: Front Immunol. 2023 Oct 2;14:1270414. doi: 10.3389/fimmu.2023.1270414 (PMC10580809; doi:10.3389/fimmu.2023.1270414)
Supplement: Supplementary file 5 [file Table_1.docx]

**Supplementary Table-1.** List of hamster gene primers used in qPCR analysis.

| **Target Gene** | **Forward primer (5'…3')** | **Reverse primer (5'…3')** |
| --- | --- | --- |
| CRP | TGGGACATTGTGCTGTCTCC | GGTTGTCTCAGGGCTCACAA |
| TNF-α | GGTTTACTCCCAGGTTCTCTTC | GGACAGGAGGTTGACGTTAT |
| IL-1β | AAAGCCTTGACCTGAGCTATC | CTTCTCCACAGCCACAATGA |
| IL-6 | GACTTCCATCCACTTGTCTTCT | TTGGGAGTAGTGTCCTCTGT |
| MIP-1α | CCTCCTGCTGCTTCTTCTATG | TGCCGGTTTCTCTTGGTTAG |
| IL17-RA | CCTGTGGGTGTATGGCTTTAT | TGGAGTCATCACCGTGTTTC |
| IP-10 | GGACTCAAGGAATCCCTCTTTC | GACTTGCAGGAATAATTTCCAGTT |
| CXCL12 | TTTGTAACTCGCCCTTCCCTC | TCTTCAAAGCTGAGCCGACA |
| CCR1 | GTGACTCCACTCCATGCCAA | GCATGAGCACCAAAACCACC |
| IL-10 | AGCGCTGTCATCGATTTCTC | CGCCTTTCTCTTGGAGCTTAT |
| IL-4 | GAAGAACTCCACGGAGAAAGAC | GGGTCACCTCATGTTGGAAATA |
| p35 | GAGACTGCTTCCCTAACAAGAG | CTGTCCGGTACATCTTCAAGTC |
| MMP-2 | CACAGGGCAGTGGGATACAG | CGCGGCACGATAGAAAACTG |
| MMP-9 | ACGACCAATTCTTCTGGCGT | CTGGCTCCTTTCCCTCTTGG |
| MMP-13 | TGTTTTCCCTCGAACGCTCA | GGTGAAAGTCAGAGGCGTGA |
| TIMP2 | CCTCTTGACGTGCTCTGGTT | ACGCCATGCCATACTTTCCA |
| TIMP3 | GAATCCCAAAGGTGGGGAGG | TGGAAGATGGCCCAGGGATA |
| TGF-β1 | TGTACAACAGCACCCGAGAC | ACTGCTTCCCGAATGTCGTT |
| COLIA1 | TGCAGTTGGTGCTAAGGGTG | GAGCACCAGCAATACCAGGA |
| TAGLN | CCCTTTAAACCCCTCACCCAG | GCAGAAAGGCTTGGTCGTTC |
| MYH11 | GTGGGTGCCAACATCGAAAC | TTTTCCTTGGCTCCAGCGAT |
| SMAD2 | CTCAACACTTTGCAGTGGGC | GAAGGTCAGTCCACCTTGGG |
| GMCSF | CATTGTGGTCTGTAGCTTCTCT | CAGGTTTCTCAGGGCTTCTT |
| GCSF | CTGGTCTCTGTCACCTCTCT | TTCCTGGTCTGGATCTTCCT |
| FGF2 | CCTTGCTATGAAGGAGGATGG | CTTCCTGGACCGGTAAGTATTG |
